# Calculation of silent substitution stimuli for full-field electroretinography using the SilentSubstiTutor application

**DOI:** 10.1007/s10633-026-10090-1

**Published:** 2026-03-14

**Authors:** Cord Huchzermeyer, Jan Kremers

**Affiliations:** Department of Ophthalmology, University Hospital Erlangen, Schwabachanlage 6, 91054 Erlangen, Germany

**Keywords:** Electroretinography, Silent Substitution, Photoreceptors, Cone fundamentals

## Abstract

**Purpose:**

The silent substitution technique allows photoreceptor directed stimulation (i.e., the selective stimulation of different photoreceptor types or a specific combination of photoreceptor types).

**Methods:**

Calculation of these stimuli is not trivial, requiring complex matrix calculations based on specific datasets (spectral power distributions of primary lights, photoreceptor fundamentals, and optical densities of pre-receptoral filters). Several tools have been published that facilitate these calculations, including an excel file and a python library, but these are difficult to use without prior knowledge.

**Results:**

We introduce an online application that allows calculation of silent substitution stimuli in a graphical user interface (GUI) for common use-cases (3 to 5 primary lights, periodic stimuli modulated around an average setting of the test field, 10° standard observers).

**Conclusion:**

The goal is to provide a practical tool that can be used in these cases, but that can also be used as a teaching tool for beginners who plan to use the more sophisticated methods.

## Introduction

Silent substitution allows targeting distinct photoreceptor classes in psychophysical or electrophysiological tests by varying the spectral composition of a visual stimulus [1, 2]. This is frequently accomplished by using devices with at least three [3], but preferably four [4] or five spectrally distinct light sources [5]. Excitation is kept constant in the non-targeted (‘silenced’) photoreceptor classes by balancing photoreceptor excitation increases, caused by some primaries, with decreases induced by the remaining primaries, considering the different spectral sensitivities of the photoreceptor classes at the corneal level (called “fundamentals”) and making use of the principle of univariance [6], which states that the excitation of a photoreceptor depends only on the number of photoisomerizations and not on the spectral composition of the light that caused them [7]. When this principle is applied to temporally modulated stimuli, all photoreceptors will detect light, but only one class will detect changes in light intensity over time [8].

Powerful tools for calculating silent substitution stimuli have been published recently [9, 10], but they are complex (due to the multitude of possible devices and stimuli) and, therefore, difficult to use without extensive technical knowledge. The SilentSubstiTutor was designed as a simple application with a graphical user interface (GUI) to create periodic photoreceptor-directed stimuli for recording ERGs using a full-field stimulator. This application was designed specifically to accompany the ISCEV extended protocol published in this issue [add Reference to Extended Protocol]. An extensive knowledge of the theories and mathematics of silent substitution is not necessary.

Based on the principles underlying silent substitution, a device can only control the modulation of the excitation of the same number of photoreceptor classes as there are primaries [2]. The excitation modulation of additional photoreceptor types can be calculated but a silent substitution can generally not be achieved. If these photoreceptor types need to be silenced, then desensitization through adaptation can possibly be employed. For simplicity, the SilentSubstiTutor APP ignores rod-driven activity and melanopsin driven activity of the intrinsic-photosensitive retinal ganglion cells (ipRGCs) when a device has only three primaries, and it will ignore ipRGC activity when a device has four primaries. Although many design decisions in the APP stem from its main purpose of creating periodic photoreceptor-directed stimuli for full-field ERGs, it can also be used to calculate stimuli for experiments with smaller test fields or for psychophysical experiments. Applications other than full-field ERGs, require careful interpretation of their results with regard to the underlying assumptions.

## Technical details of the app

The *SilentSubstiTutor*-App is written in R, a language for statistical computing [11], using the *shiny* framework for creating a web-based graphical user interface [12]. The source code is hosted on GitHub (https://github.com/huchzi/silentSubstitutor) under the MIT license, so that everyone has the right to read, copy, modify the local copy and suggest integration of the modifications into the main repository using “pull requests”. The GitHub repository includes a version history (see https://github.com/huchzi/silentSubstitutor/blob/main/CHANGELOG.md).

The colorSpec package is used for interpolation of spectral data, including those imported by the user, and for some calculations [13].

## Disclaimer

Although the calculations implemented in this application have been carefully validated, ISCEV takes no responsibility for erroneous calculations and it is recommended that results are checked against manual calculation, particularly in situations where the accuracy of results is of critical importance.

Furthermore, the interpretation of the results depends on several assumptions that are rarely fulfilled completely in practice and may not be valid in certain circumstances. Consequently, clinical and scientific interpretation of the results requires a critical appraisal of these assumptions.

As with other ISCEV standards and guidelines, this application does not encompass clinical safety standards or standards for clinical care and management. For clinical use, instruments approved for medical application must be employed, and local regulations regarding clinical safety, patient care, and data protection must be strictly observed.

## Running the app

The APP can either be run on a local R installation (accessed using a browser), or it can be accessed online. For local installation, R can be downloaded for different operation systems from the *Comprehensive R Archive Network* (CRAN, https://cran.r-project.org/). At the R command line, the S*ilentSubstitutor* package can be installed using the *devtools* package (for detailed instructions see S1). After loading the package, the APP can be run in the R command line. Then, the APP can be accessed with the system browser. If the RStudio IDE is used, a browser will be opened automatically.

Please find the link to the APP either via the ISCEV homepage (https://iscev.wildapricot.org/standards) or via the github repository (https://github.com/huchzi/silentSubstitutor/blob/main/Readme.md).

## Underlying spectral data

Several spectral datasets are necessary for the calculation of silent substitution stimuli. These datasets were downloaded from the Color Vision Research Lab (http://www.cvrl.org/; range: 390–780 nm, 2 nm steps). For modelling a standard observer, the photopic luminous efficiency function V_lambda_ [14] and the spectral sensitivities of the photoreceptors are required. The SilentSubstiTutor APP is based on the Stockman and Sharpe 10° cone fundamentals for L-, M-, and S-cones [14, 15], the scotopic luminous efficiency function [16] for rods, and the CIE melanopsin fundamentals [9, 17]. For calculating the stimuli, these spectra cannot be modified for the individual observer.

However, fundamentals can be modified for estimating the consequences of individual deviations, including pre-receptoral filters. The 10° cone fundamentals assume a macular pigment optical density of 0.3 and a lens age of 40 years [18]. Modification of the fundamentals is based on the macular pigment optical densities provided by Stockman, Sharpe and Fach [15]. Lens ageing is modelled using the lensAbsorbance() function of the colorSpec package, which is based on the data by Pokorny, Smith and Lutze [19].

## Overview of calculation

The creation of a stimulus consists of three steps performed in separate panels:

1. description of the instrument and its settings,
2. calculation of the stimulus and check for technical feasibility
3. validation of the stimulus.

In the first step, the emission spectra and the luminances [cd/m^2^] of the primary lights are selected. In the second, desired photoreceptor contrasts (defined as the Michelson contrast of the modulation of photoreceptor excitation) are entered by the user and stimuli are calculated by the APP in the second step. In the third step, the underlying spectral datasets are modified, so that errors caused by deviations from the underlying assumptions can be estimated.

## Step 1: set up visual stimulator device

In the first panel, the emission spectra of the most common primaries can be chosen from predefined spectra using the dropdown list. Alternatively, when the spectra are known (e.g. through direct measurements with a spectroradiometer), they can be uploaded in the „Advanced Settings “. Furthermore, the mean luminance (in cd/m^2^) of each primary should be set.

The number of primaries shown depends on the number of columns in the selected spectra. The names of the primaries will be taken from the imported files, while the colors in the plots are derived by approximating the colors of the primaries in RGB space based on the emission spectrum.

**Fig. 1:**
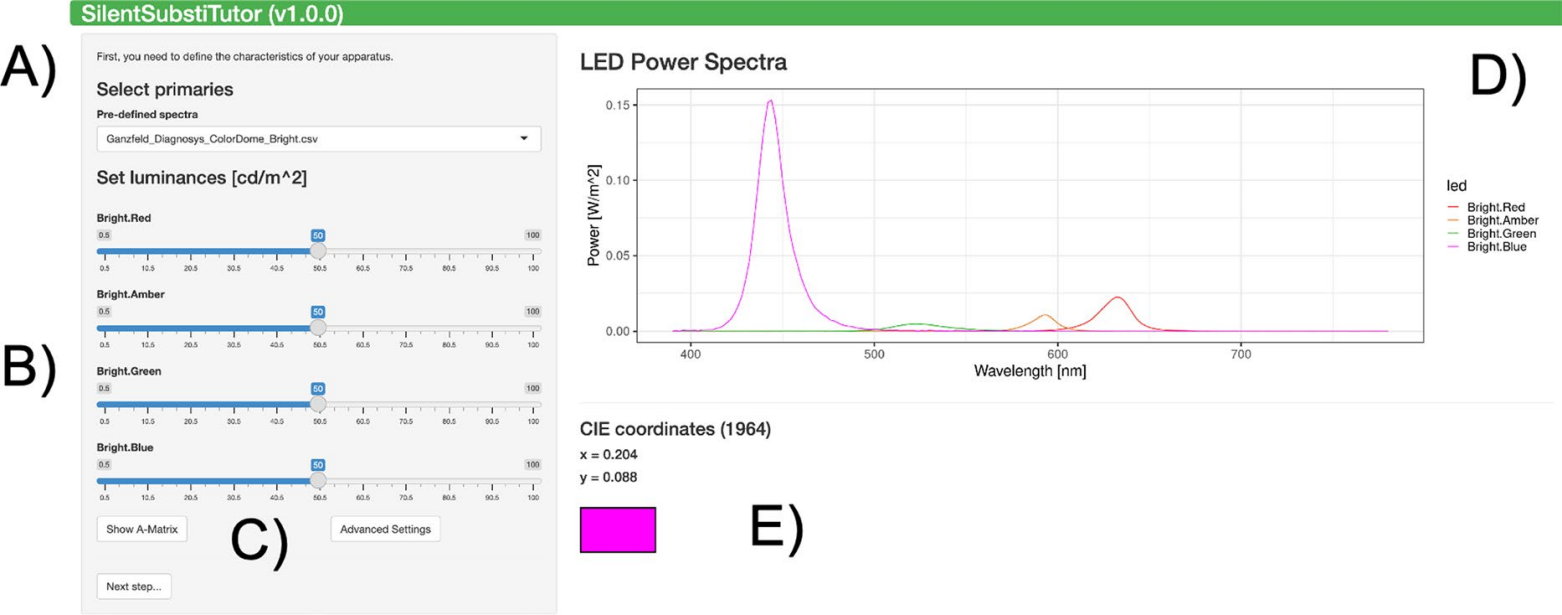
Panel 1: Setting up the visual stimulator. **A** Select emission spectra (custom spectra can be imported under „Advanced Settings “, **B** set the luminances of the primaries, **C** check A-Matrix, set luminance ranges and bookmark settings, **D** check LED power spectra, and **E** check CIE coordinates

The APP will normalize the emission spectra of the LEDs to a radiance area-under-the-curve of 1 W/(m^2^.sr) and scale it according to the luminances selected in the sliders. Thus, the unit of the emission spectra is not important. The right side of the panel show the resulting spectral power distribution (W/(m^2^.sr.nm)) and CIE64 coordinates. During the experiment the stimulus will be modulated around these settings. The hue that is shown in the box (Fig. 1E) along with the CIE64 coordinates approximates the mean chromaticity of the stimulus in situ*.* Slight deviations may occur.

The APP assumes that the emission spectra of each primary is the same for all mean luminances and that *the* actual output luminance is correctly defined by the nominal luminance. For most devices, these assumptions hold. Actual and nominal luminances may differ when they are close to zero (threshold effects) or near the upper output limit (saturating effects). No primary can be set to zero luminance, because that would correspond to a device with n-1 primaries. In this case, a lower number of primaries should be chosen. PySilSub and the STAR protocol offer more flexibility in this regard. The information needed about the visual stimulator to be used are found in Table 1.

**Table 1:** Parameters describing the stimulator device

Parameter	Comment
Number of primaries	3–5, cannot be manually selected; inferred from the emission spectra file
Emission spectra	CSV/Excel file with wavelengths in 2nm steps and corresponding intensities [W/m^2^]
Maximally possible luminance	[cd/m^2^]
Luminance resolution	Negligible if sufficiently high

The LED luminances set here must be chosen carefully, because they determine the available photoreceptor contrasts. For determining the optimal luminances, it is recommended to consider the emission spectra. It is a good starting point to set the small and medium wavelength primaries to slightly lower luminances, and the long wavelength primaries to slightly higher luminances. Usually, the short wavelength primaries must be set to relatively low luminances, because this still results in relatively high radiances. Generally, settings of red: 80cd/m^2^, amber: 100cd/m^2^, green 40cd/m^2^ and blue 4cd/m^2^ yield feasible rod and cone contrasts. If sufficient contrasts cannot be reached with these settings, the plots showing LED luminances as a function of time can be considered, to determine which LED limits the contrast, i.e. which LED has a contrast of 100%. Chose new settings so that this LED has a higher luminance. Changing luminance of all LEDs by a multiplication or division by the same factor will result in identical photoreceptor contrasts. The STAR protocol offers quantitative methods for optimizing this setting.

## Advanced settings

Spectra for several devices are available in the app. To import spectra, an excel or a csv file can be uploaded in the “Advanced Settings” panel. It is also recommended to set the ranges for the luminance sliders in this panel, because these settings will be used to warn about luminances that exceed the upper limit of luminance for the device. When the device is completely described, the “Bookmark” button in the “Advanced Settings” panel can be used to save the settings with a browser bookmark, so that they will be immediately available when the APP is started.

## A-Matrix

The calculation of stimuli follows previously published methods [3, 9, 20]. Briefly, a squared A-matrix is calculated by matrix multiplication of the emission spectra (in W/(m^2^.sr.nm)) and the photoreceptor fundamentals of the photoreceptors that are controlled during the experiment. Solving the equation is only possible for squared A-matrices (i.e., with equal numbers of rows and columns), but a separate A-matrix is used to include the contrasts in the photoreceptors that are not controlled. For calculation of contrasts, the A-matrix is normalized so that all columns sum up to 1. In the next step, the inverse of the A-matrix is used for calculating the contrasts in the primaries’ luminances. For this, a vector, that includes the desired photoreceptor contrasts, is employed. Please observe that the desired photoreceptor contrast is zero in a silent substitution condition.

## Panel 2: calculate stimuli

In this step, the user must provide the desired photoreceptor contrast. Then, the contrasts in each primary, that are needed for the wanted condition, are returned by the APP. These contrasts may exceed 100% and are technically not feasible. Then they should be scaled to the maximal technically feasible contrasts. A final check, that the resulting primary contrasts are technically feasible, is required.

In the app, the desired photoreceptor contrasts can be selected by using the text input fields on the left. The contrasts of more than one photoreceptor type can be non-zero. Negative contrasts can be chosen, indicating counterphase modulation with those photoreceptor types that have positive contrasts.

Stimulus and photoreceptor contrasts are proportional, meaning that a multiplication of stimulus contrasts in all primaries by the same factor will lead to the multiplication of all photoreceptor contrasts by the same factor. By pressing the “Maximize” button, the contrasts in the primaries (and thus in the photoreceptors) are rescaled so that the contrast is 100% in one primary and less in all other primaries.

The APP will calculate a stimulus and show the results in the top right corner. We have validated the calculations against the PySilSub package and against a spreadsheet used in earlier publications [10, 20]. Contrasts exceeding 100% are shown in red. Furthermore, the *MinLuminance* and *MaxLuminance* values [cd/m^2^] will be highlighted when these values exceed the luminance range set for the primary stimuli. This warns the user that the stimulus is technically not feasible. Very small primary contrasts should be avoided, because the resolution of the used device may not be sufficient.

## Export of data

**Fig. 2:**
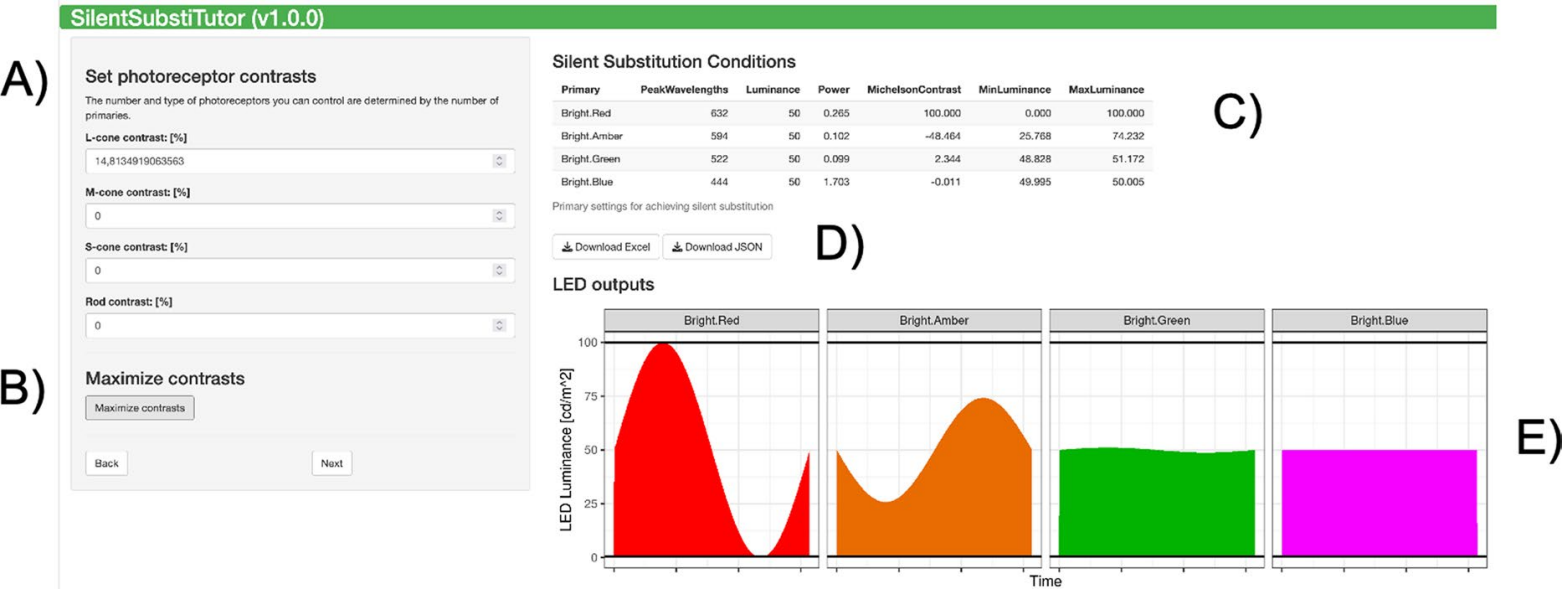
Panel 2: Calculating stimuli. **A** Set the desired photoreceptor contrasts. If more than one photoreceptor type should be stimulated, the ratio is more important than the absolute values at this point. These can be calculated at a later stage. Negative values signify stimulation that the modulation is mirror imaged (and in counterphase for sinusoidal stimuli). **B** The contrasts can be rescaled to the maximally possible contrasts (where one primary has 100% contrast and all other have contrast that are less or equal to 100%). **C** This table shows the resulting contrast settings for each primary, along with the minima and the maxima that are reached. These MinLuminance and MaxLuminance values [cd/m^2^] are highlighted in red if they exceed the range settings in panel 1. **D** The table can be downloaded as Excel or JSON files. This can be used for calculating files that drive the stimulus device. **E** illustrates the modulation of the primaries. The range limits are shown as horizontal black lines

Currently, the result table can be downloaded as an excel sheet or as a JSON file (see Fig. 2D). This can be used to facilitate implementing these stimuli on the device. In the future, scripts can be created for automatic conversion.

## Panel 3: validate stimuli

**Fig. 3:**
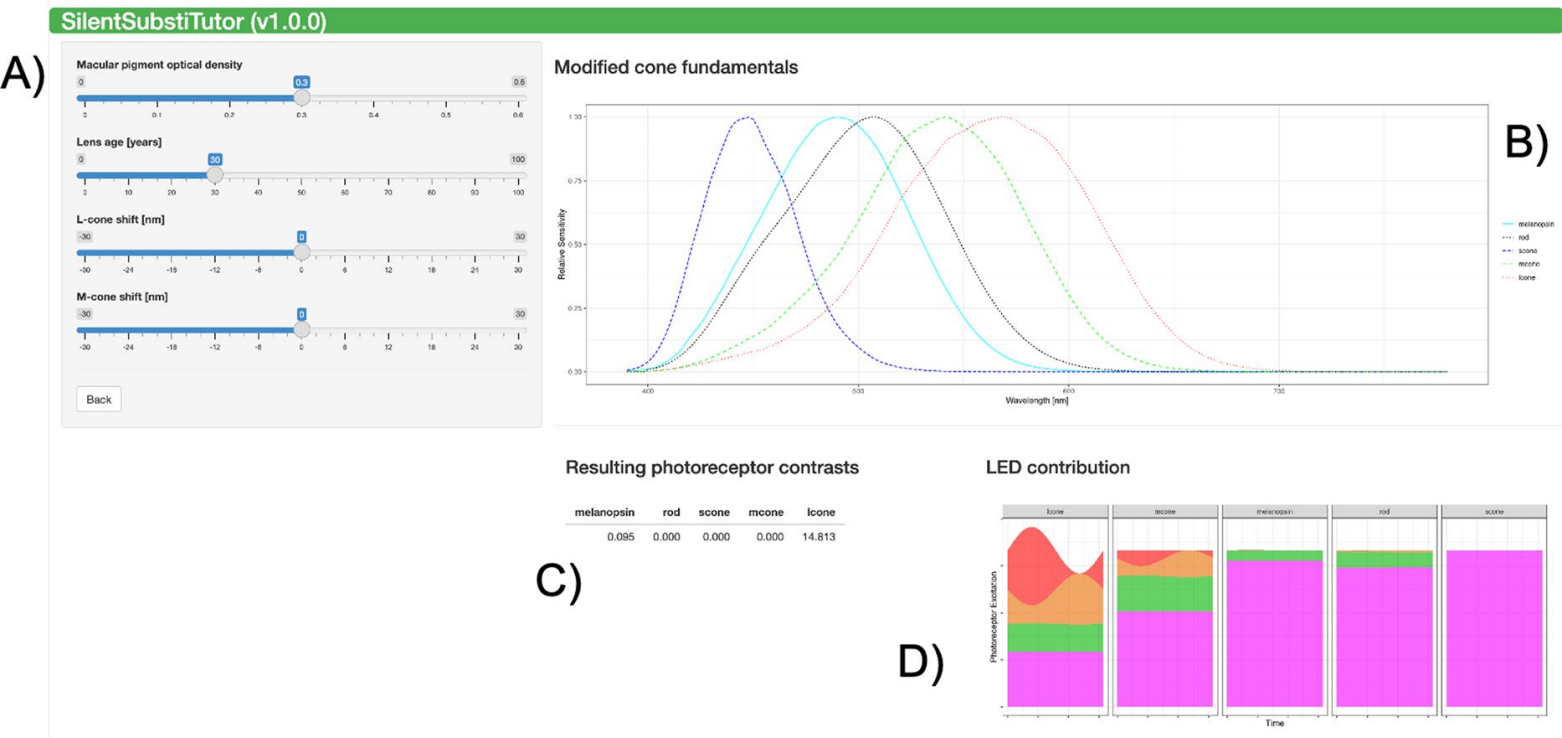
Panel 3: Validation of stimuli. **A** The different physiological values that influence the individual photoreceptor fundamentals can be modified. **B** shows the resulting modified fundamentals. **C** shows the resulting photoreceptor contrasts when using these modified fundamentals along with the calculated contrast settings from Fig. 2C. These can be used to adjust the stimulus settings. If no parameters were modified, this table can be used to check the contrasts in the photoreceptors that were ignored during calculation for devices with three or four primaries. **D** An educational plot that shows how the modulation in each primary influences the photoreceptor excitation

In the final panel, the stimulus can be validated (Fig. 3). When using a three- or four-primary device, the modulation of the photoreceptors that were ignored in the calculations should be checked. The assumptions of silent substitution are never perfectly met, due to the inter- and intra-individual variability in pre-receptoral filtering (lens density, macular pigment, and retinal blood vessels). The SilentSubstiTutor APP does not support direct correction for these factors, but it allows estimating the consequences of lens ageing, macular pigment optical density variation and shifts in the spectral sensitivity curves of L- and M-cones, for example due to single nucleotide polymorphisms in the LWL and MWL opsins. This can be done by adjusting the sliders on the left.

Note that the photoreceptor responses are given rather than their excitation modulation. Recorded ERG amplitudes may depend on many factors including the state of adaptation and the temporal frequency. If the adaptation sensitizes the photoreceptor class that was intended to be silenced, residual responses may still result in significant responses. On the other hand, other factors can be used to minimize intrusion of photoreceptor classes that cannot be controlled. For instance, when using a three primary stimulator, rods and ipRGCs cannot be controlled, their influence can be minimized by using photopic conditions (thereby desensitizing the rods) and high temporal frequencies (above the flicker fusion frequency of ipRGCs and possibly of rods).

Although the calculations are primarily intended for ERG recordings, they can be also used for other type of measurements, such as psychophysics. Then it should be considered that the effects of retinal eccentricity may be different from those on the ERG.

## Conclusions

The SilentSubstiTutor is a simple, user-friendly application for calculating photoreceptor-directed stimuli using the silent substitution paradigm. It was primarily designed for creating periodic stimuli to be used in full-field ERGs as described in the ISCEV extended protocol [insert reference], but the calculated contrasts can easily be adapted to similar settings. In comparison to currently available tools for calculating silent substitution stimuli, the stimuli are more restricted, but it is much easier to learn and to apply them. In the future, this will enable more widespread application of the silent substitution technique.

## Data Availability

No datasets were generated or analysed during the current study.
